# An Investigation of First-Year Students' and Lecturers' Expectations of University Education

**DOI:** 10.3389/fpsyg.2017.02218

**Published:** 2018-01-26

**Authors:** Stefanie Hassel, Nathan Ridout

**Affiliations:** ^1^Department of Psychiatry, Cumming School of Medicine, University of Calgary, Calgary, AB, Canada; ^2^Department of Psychology, School of Life and Health Sciences, Aston University, Birmingham, United Kingdom

**Keywords:** student expectations, lecturer expectation, UK higher education, University education, teaching styles, teaching experience

## Abstract

Transition from school to university can cause concern for many students. One issue is the gap between students' prior expectations and the realities of university life, which can cause significant distress, poor academic performance and increased drop-out rates if not managed effectively. Research has shown several similarities in the expectations of staff and students in regards to which factors determine academic success, but there is also evidence of dissonance. For example, staff consider independent study and critical evaluation as key factors, whereas students view feedback on drafts of work and support from staff as being most important. The aim of the current study was to determine what expectations students hold when starting university education, and what expectations university lecturers have of students entering university. Lecturers (*n* = 20) and first year students (*n* = 77) completed a series of questionnaires concerning their expectations of learning in HE (staff and students) and their approach to teaching (staff). Results revealed that students have largely realistic expectations of university. For example, the majority expected to be in charge of their own study. Some unrealistic expectations were also evident, e.g., most expected that teaching would be the same at university as it had been at school. The expectation that lecturers would provide detailed notes varied as a function of student age. Lecturers reported modifying their expectations of students and adapting their teaching approach according to year of study. Information-transmission/teacher-focused style was more common when teaching 1st year students; a more concept-changing/student-focused approach tended to be used when teaching 2nd year students (and above). Lecturer's expectations of student engagement did not differ according to year. Less experienced lecturers reported more negative expectations of student engagement than did experienced lecturers. In line with previous work, we observed overlap in expectations of staff and students, but some clear differences too.

## Introduction

Transition from school/college to university can be extremely challenging, both for the student and academic staff involved in teaching the new cohort. This transition has been identified as a major cause of anxiety amongst first-year university students (Lowe and Cook, 2003). Failure to successfully manage such transition may result in significant distress, poor academic performance, and increased drop-out rates (Yorke and Longden, 2004). It is notable that the transition to university may be particularly difficult for mature students with families, for students who are the first generation to go to university, and for students who come from ethnic minorities that are underrepresented in a student population (Briggs et al., 2012). Since the arrival of the Teaching Excellence Framework (TEF) this has become particularly relevant for the UK Higher Education (HE) sector. TEF recognizes institutions which do the most to encourage students from a range of backgrounds, and provide support to facilitate their retention and progression.

According to Smith and Wertlieb (2005), a key factor in the ease of transition from school or college to university is student expectations, or, more specifically, the gap between students' prior expectations of HE and the reality of university life. There is a growing body of evidence showing that many students arrive at university with unrealistic expectations (Lowe and Cook, 2003; Smith and Hopkins, 2005; Crisp et al., 2009; Murtagh, 2010; Kandinko and Mawer, 2013). For instance, incoming students often overestimate the amount of contact time with staff that will be offered at university (Smith and Hopkins, 2005); they also have unrealistic beliefs about class sizes, staff availability, and work-load that are inconsistent with reality (Lowe and Cook, 2003). With this in mind, students often arrive ill-prepared for studying at university, where teaching regularly takes place in large class sizes, where students are taught by staff who are involved in a variety of other roles in addition to teaching, and where the emphasis is on independent learning. As noted by Murtagh (2010), the transition from the highly controlled, teacher-driven learning environment of schools or colleges to university, where the student is responsible for their own learning, is perhaps the biggest challenge for the student. Furthermore, such mismatch between a student's expectations and reality has the potential to color their experiences during first year. This is important, because first year experiences play a significant role in shaping students' attitudes and performance in subsequent years (Tinto, 1993). Additionally, the initial weeks of first year are also the time at which students are most likely to drop out from their course (Smith and Hopkins, 2005).

To manage the transition of students into university successfully, universities need to be proactive in working to minimize any potential discrepancies between what students expect of university (and by proxy, of their lecturers) and what, in turn, is expected of them. This issue is likely to become increasingly important given the proposed changes to tuition fees and the increasingly consumerist ethos within the HE sector, particularly against a backdrop of continued expansion of student numbers at UK universities.

### Student expectations of studying at university

A common expectation of students is that a university education will enhance their academic and vocational prospects, but also provide opportunities to become independent and to enjoy themselves (Lowe and Cook, 2003; Kandinko and Mawer, 2013). Employability has become a key issue in the UK HE sector, particularly since the increase in student fees at British universities (Kandinko and Mawer, 2013). The career-focused approach to education can be beneficial. For example, Tinto (1987) reported that students who were more certain about their long-term career goals were more likely, and faster, to successfully transition to university than students who studied without clear career trajectories. However, this focus on employability can potentially lead to a shallow approach to learning, with students concentrating on merely passing assessments to get through the course rather than developing a deep approach to learning and trying to understand the course material.

Aside from expectations concerning improved employment prospects and increased personal independence, students generally come to university with few expectations and with little notion of how to be successful; they often view it as a continuation of secondary school. For example, Lowe and Cook (2003) reported that nearly a third of their cohort of first year students expected that lecturers would use similar teaching styles to those they had experienced at school. Thus, students found themselves unprepared for the more relaxed and informal style of teaching they encounter at university. With regards to expectations about the style of teaching that students may encounter at university, there was an interesting distinction reported by Lowe and Cook (2003). A “*significant”* number of their cohort reported receiving much more detailed material in, or for, class than they had expected, whereas an equally large number of students found that the lecture material was not as detailed as they had expected (Lowe and Cook, 2003). These variations are likely to make it harder for universities to manage students' expectations during the transition period into HE.

Another issue of note concerns students' expectations of how they will be taught at university. For example, Kandinko and Mawer (2013) reported that students exhibited a preference for small tutorial-style classes, as opposed to larger lecture-type classes. This is because the former offers greater opportunities for face-to-face interactions with teaching staff. However, the rapid expansion of the HE sector has seen a movement toward greater reliance on large lecture-style classes to deliver course material rather than small group teaching (Crosling et al., 2009), especially during 1st year. Incoming students often overestimate the amount of contact time that they will be offered at university (Smith and Hopkins, 2005) and can have expectations about the role of teaching staff that are inconsistent with the reality of studying at university. For example, although HE staff tend to consider the responsibility for learning to be primarily the students' responsibility (Crabtree et al., 2007), some students tend to consider that lecturershave the greater responsibility for students' learning (Killen, 1994). On the other hand, Crisp et al. (2009) demonstrated that students' expectations can be consistent with those of staff, as their cohort recognized that their success at university would be primarily be their own responsibility. Despite the evidence of congruent staff/student expectations it remains the case that there are often discrepancies between the students' expectations of the role of staff and the reality of university life. For example, Lowe and Cook (2003) reported that 41% of their cohort had expected staff, i.e., lecturers, to be more sympathetic and reassuring, and 35% had thought that lecturers would be more helpful and friendly. This is important because, expectations of positive staff-student interaction and mutual understanding seem to be vitally important for students' successful transition into university (Clark and Ramsay, 1990; Grosset, 1991; Johnson and Watson, 2004; Keup and Barefoot, 2005), as is lecturers' involvement in facilitating academic and social integration. Negative perception of academic staff has been shown to adversely impact students' chances of success (McInnis et al., 1995; Maxwell, 1996; Lizzio et al., 2002).

The most significant difference, or gap, between what students think university is like or what they expect from university, relates to their preparedness academically, i.e., their expectations of potential academic difficulties they may encounter. Although some studies have reported that students were rather confident about their abilities to cope with academic requirements (Cook and Leckey, 1999), others have reported that students expect to struggle with the demands of learning in HE. For example, Lowe and Cook (2003) reported that two-thirds of their sample expected to experience problems in coping with the academic demand. Interestingly, upon follow-up, it turned out that only 50% of students actually experienced academic struggles. Thirty-nine percent of students shared that they had struggled to keep up with the workload and over a third reported that they experienced difficulties in developing an independent learning/study style, i.e., being responsible for their own learning. These issues are likely related to students' expectations prior to arriving at university. For example, Lowe and Cook (2003) reported that, on entering university, 57% of their cohort did not know how much studying, including attending classes and independent reading, would be required per week. Indeed, students often underestimate the number of hours of independent study that would be required for their course (Crisp et al., 2009) and were unprepared for this aspect if university life (Murtagh, 2010). Murtagh (2010) also highlighted that students arrive at university without a clear understanding of how they are going to be assessed, supporting Lowe and Cook's (2003) observation of nearly 20% of their sample not knowing about assessments on their course. There is evidence that students may harbor unrealistic expectations about assessments, for example, supposing that lecturers will provide detailed feedback on drafts of their work and that staff will be able to return assessed work within a week (Crisp et al., 2009).

Students expect to, and often do, experience financial difficulties during their degree. For example, Lowe and Cook (2003) reported that 45% of the cohort they studied experienced financial hardship. With this in mind, students often expect to combine paid work with their studies. Crisp et al. (2009) observed that 70% of their cohort expected to be doing some form of paid work alongside their degree. Longden (2006) showed that over 40% of their sample of first-year students were working alongside their studies, with 10% of the sample working more than 20 h per week. The need for students to undertake paid work has been implicated in rates of non-attendance at lectures, which is a growing problem in HE (Cleary-Holdforth, 2007; Field, 2012). This is interesting because students recognize that attendance at lectures and other teaching sessions is important for their academic performance (Crisp et al., 2009). Given that missing lectures and teaching sessions can disadvantage students, universities have responded by providing additional resources, such as offering notes and/or recorded lectures, which can be accessed online. These are popular with students, but the concern remains that they might exacerbate the problem of non-attendance (Grabe, 2005; Chang, 2007; Karnad, 2013).

An issue that needs to be considered is that where students have few or inaccurate perceptions of university education prior to undertaking undergraduate study this may contribute to a disengagement from the educational and social aspects of university life. Such disengagement can have detrimental effects on students' academic performance, their personal and social development, and may also affect student retention (Lowe and Cook, 2003). A need for better preparation, aided by appropriate communication between teachers and students and between secondary and tertiary educational institutions, is obvious. Universities too need to offer appropriate academic, attitudinal, and social preparation courses for incoming students. This should be a process, rather than an event and, in addition to academic preparation, linked to peer-mentoring and staff-student interaction opportunities (Lowe and Cook, 2003).

### Lecturers' expectations of university students

There is a paucity of research assessing what lecturers expect of students when they first enter university and very few studies have investigated the perceptions of both students and lecturers regarding factors that influence academic success (Killen, 1994; Fraser and Killen, 2003). Fraser and Killen (2003) showed that, overall, there was considerable agreement between the responses of first-year students and lecturers about which factors impact on academic success. However, students and lecturers significantly differed on the importance placed on “regular attendance at lectures.” Students did not expect having to attend all lectures, or considered irregular lecture attendance to affect their academic success. Lecturers expected students to regularly attend lectures and linked attendance with success (Killen, 1994; Fraser and Killen, 2003). In the context of essay writing, McEwan (2015) reported several interesting differences between the expectations of staff and students. For example, 64% of their sample of HE tutors considered that the lecturer is the target audience for an essay, whereas only 38% of their student sample thought this was the case. Also, 71% of staff thought that students should critique their sources, whereas only 25% of the students thought this was necessary.

With regards to expectations that would contribute to students' academic failure, there was significant disagreement between lecturers and first-year students. Students attributed external causes to less successful academic performance, specifically part-time work. Lecturers, on the other hand, thought that it was “inadequate and/or poor exam preparation” that led to students' academic failure, i.e., more internal characteristics (Fraser and Killen, 2003). Additionally, there was a tendency for blame-attribution: students tended to blame lecturers for academic failure yet lecturers held the students themselves responsible for not achieving to the best of their abilities (Killen, 1994; Fraser and Killen, 2003). According to Mischel (1973) lecturers expect students to be independent learners by the time they enroll at university, but this assumes that incoming students already understand the need to be efficient in balancing their desire for achievement with a strong sense of purpose and enjoyment from academic activities. Fraser and Killen (2003) reported that lecturers also expect students to be self-disciplined and self-motivated.

Recent changes in student fees have led to an increasingly consumerist ethos amongst the student population which has influenced students' expectations (Kandinko and Mawer, 2013). The question remains as to whether staff expectations of students has also been influenced by these changes. The match, or mismatch, between student and staff expectations is important, as it can have implications for students' academic performance, but also their social and emotional wellbeing (Williamson et al., 2011). With this in mind, it is important to gain information about the current match or mismatch between students' and staff expectations.

The aim of the current study was to determine the expectations of incoming first year students and the academic staff who teach them and to establish the relative match—or mismatch in the expectations of these two groups. Students were presented with a questionnaire, based on Lowe and Cook (2003), that assessed their expectations of the academic and social aspects of starting at university. Lecturers were presented with the Approaches to Teaching Inventory (ATI; Trigwell and Prosser, 2004), which assesses whether lecturers adopt more of an *Information-Transmission-Teacher focused (ITTF)* approach; or more of a *Conceptual-Change-Student focused (CCSF)* teaching style and with statements reflecting positive and negative student engagement.

## Methods

### Participants

Data were available for 77 students enrolled in either the Single Honours Psychology Programme or a Joint Honours Degree Programme with Psychology being one of the two subjects studied. Additionally, data were collected from 20 staff members who are currently lecturing on the Psychology Programme at Aston University, Birmingham, UK. All participants were recruited over a period of ~2 months, between October and November 2014. The experimental protocol was explained to participants and written informed consent was obtained, in accordance with the Declaration of Helsinki. Ethical approval was obtained from the Centre for Learning Innovation & Professional Practice (CLIPP) at Aston University, Birmingham, UK prior to data collection.

#### Student sample

The mean age of the student participants (*n* = 77) was 19.1 years (*SD* = 3.0 years), with a range of 21 years: minimum age: 18 years; maximum age: 39 years. The sample consisted of 15 men (19.5%) and 62 women (80.5%)—this male:female ratio is characteristic of the undergraduate psychology programme at Aston University. Seventy-three participants (94.8%) were studying on the Single Honours Psychology Programme, the remaining participants (5.2%) were studying on the Joint Honours Degree Programme. The average entry tarif for this cohort was 380 UCAS points (Guardian University Guide, 2015), which is consistent with the average of 386 over 5 years (2012–2017) and 87% of the cohort progressed into second year, which is consistent with the average progression rate 86% over 5 years (2012–2017). However, only 4% of the cohort actually withdrew or were withdrawn from the programme, which is slightly lower than the average withdrawal rate of 6% over 5 years (2012–2017). The majority of participants (73; 95.8%) were in their first-ever degree programme; the remaining participants (5.2%) had previously entered a degree programme without completing it.

#### Staff sample

Data for lecturers (*n* = 20) showed that 10 lecturers' responses (50%) for the questionnaire were concerning first-year students, six lecturers' responses (30%) were concerning second-year students and four lecturers' responses (20%) were concerning third/final-year students. On average, lecturers had been teaching 14.5 years (*SD* = 9.1). The sample included novice and experienced lecturers with a teaching-experience range of 39 years (minimum years teaching: <1 year; maximum years teaching: 40 years). The course for which the questionnaire was completed was taught—on average—for 4.2 years (*SD* = 4.4 years; range: 19 years; minimum years teaching on this module: <1 year; maximum years teaching on this module: 20 years).

### Measures

Students completed a questionnaire that was created specifically for this study but which was based on the survey used by (Lowe and Cook, 2003). The questionnaire assesses students' expectations of the academic and social aspects when starting at university, and is comprised of three sections: (a) *Reasons for Attending University* (15 items); (b) *Academic Aptitude* (15 items); (c) *Teaching Expectation* (15 items). Students were required to rate their agreement with each statement on the questionnaire on a 5-point Likert scale ranging from 1 (strongly disagree) to 5 (strongly agree).

Lecturers completed the ATI (Trigwell and Prosser, 2004), a 16-item self-report questionnaire which consists of two main scales: (a) reflecting an information-transmission/teacher-focused (ITTF) approach; (b) reflecting a conceptual-change/student-focused (CCSF) approach. Each scale is further subdivided into “*Intention”* and “*Strategy”* subscales. The “Intention” subscale is associated with what is meant to be achieved; the “Strategie” subscale is linked to how this would be achieved (teacher-focused; student-focused; teacher-student interaction). “*Intentions*,” thus, range from “transmission of subject content to students” to “helping students change their conceptions of the content.” Lecturers were required to rate their agreement with each of the statements on the questionnaire on a 5-point Likert scale, ranging from 1 (only rarely) to 5 (almost always). Higher scores indicate higher levels of endorsement of the assessed teaching style. Lecturers were also presented with statements reflecting positive student engagement (eight items, e.g., *They'll be interested in learning new material*) and negative student engagement (eight items, e.g., *They won't be interested in what I teach*). They were asked to indicate which of these statements they would expect from the students they teach.

### Data analysis

#### Analysis of student questionnaire

Total scores for the student questionnaire were calculated and subsequently, emerging clusters were generated. Statements reflecting students' expectations were then analyzed using One Sample *t*-tests.

Using cluster analysis, we examined which of the statements (clusters) would help to identify “similar students,” i.e., which statements would be a best and/or worst predictor of student expectations. Initial cluster centers were identified, using Agglomerative clustering, a hierarchical method to define the number of discrete clusters.

The K-Means Cluster Analysis, a non-hierarchical procedure, was subsequently applied to classify cases into groups that are relatively homogeneous within themselves and heterogeneous between each other. Then, cases were assigned to clusters based on the distance from cluster centers, using an iteration factor of 5. Finally, locations of cluster centers were re-assessed based on the mean values of cases in each cluster.

In exploratory analysis we also assessed if age of student would have an effect on questionnaire scores. Thus, students were separated into two groups, those under the age of 20 (*n* = 69) and those aged 20 and above (*n* = 8). Non-parametric Mann–Whitney *U*-tests were conducted to assess the differences between these two groups.

### Analysis of staff questionnaire

Total scores were calculated for the ITTF and the CCSF subscales of the ATI; scores were also generated for the “*Intention”* and “*Strategy”* subscales. To assess the relationship between teaching experience and teaching approaches, Pearson correlations were conducted between scores on the ITTF, ITTF-intention, ITTF-strategy, CCSF, CCSF-intention, and CCSF-strategy scales and years of teaching experiences (both, on the module selected to be the focus of the ATI and overall years of teaching experience). To account for correlations with sub-scales of the ITTF and CCSF, Bonferroni-corrected *p*-values (0.05/3 = 0.016) were used to assess significance.

Statements reflecting the positive or negative engagement of students were analyzed using One Sample *t*-tests. The testing variable reflected that at least half of the positive engagement items and half of the negative engagement items were endorsed by lecturers.

Paired sample *t*-tests also assessed if there was a significant difference between the expectations of positive or negative student engagement items. Exploratory analyses were also conducted to assess changes if the test variable reflected that all positive but no negative engagement items would be endorsed by lecturers.

One-Way ANOVAs were then used to assess group differences (more years of teaching experience vs. fewer years of teaching experience) on the endorsement of positive and negative student engagement.

## Results

### Student expectations questionnaire—summary of endorsed statements

Total scores for the student questionnaire were calculated, then emerging clusters were generated. Subscale-identified clusters are presented in Table 1. How students endorsed individual items of identified clusters is summarized in Tables 2–**4**.

**Table 1:** Themes (clusters) assessed in the student questionnaire.

**Reasons for attending university**	**Academic aptitude**	**Teaching expectation**
Ambition	Academic aptitude struggles	Expectation of Teaching being facilitating (student-focused)
Lack of other opportunities	Other struggles (Financial, Emotional, Support)	Expectation of Teaching being information transmitting (teacher-focused)
Social factors		Expectation of Learning being similar to college (high-school)
Perceived status and expectations		

**Table 2:** Summary of endorsement of items presented for *Reasons to Attend University* (in percentages); ^*^reverse score items.

**Reasons for attending university ambition, drive, motivation** I came to university because I wanted …	**Strongly disagree (%)**	**Disagree (%)**	**Neither agree/disagree (%)**	**Agree (%)**	**Strongly agree (%)**
to get a clearer idea about career decisions	13.1	4.0	7.0	58.4	29.9
to maximize my options before making career decisions	2.6	1.3	7.9	50.7	37.7
wanted to go to university (always)	1.3	1.3	11.7	50.7	35.1
and needed a university degree to get the job I want	1.3	0	28.6	26.0	44.2
**Lack of other opportunities**					
I came to university because …					
it is better than being unemployed	0	3.9	11.7	29.9	54.6
it seems like the normal thing to do	2.6	6.5	36.4	46.8	7.9
^*^I wanted to get away from home	19.5	31.2	26.0	18.2	5.0
^*^I wanted to postpone decisions about my career	14.3	39.0	18.2	23.4	5.0
**Social factors**					
I came to university because					
^*^I wanted to enjoy myself before starting work	3.9	13.0	36.4	32.5	14.3
all my friends are going to university	16.9	32.5	28.6	19.5	3.0
I wanted to find a partner	36.4	41.6	14.3	6.5	1.0
**Perceived status and expectations**					
I came to university because					
I liked the idea of going to university	1.3	2.6	2.6	64.9	28.6
this is what my parents expected of me	5.2	18.2	31.2	27.3	18.2
^*^I wanted to post-pone the need to start work	0	20.8	35.1	35.1	9.1

With regards to *reasons for attending university*, the majority of students (~60–87%) expected university to provide further information to help them make decisions about their future careers, or to start those careers. However, about 30% also acknowledged attending university to postpone career decisions. Although social factors (e.g., enjoying themselves before starting to work) formed part of university expectations for ~46% of students, peer pressure did not seem to affect university attendance, although parental expectation may have had some influence, for ~45% of students (see Table 2).

Regarding *anticipated academic struggles*, nearly 60% of students expected to struggle with their workload, nearly 50% thought the pace of teaching and subsequently learning would be too fast. However, nearly 45% of students felt confident that they understood the concept of academic teaching and learning, and despite potentially struggling with the workload, were confident in their abilities for independent and self-directed studying and learning. With regards to *other struggles*, nearly 45% of students expected to endure financial struggles, and between 40 and 50% students expected to experience emotional problems (e.g., missing family and friends) and particularly, examination anxiety (see Table 3).

**Table 3:** Summary of endorsement of items presented for *Anticipated Obstacles* (in percentages); ^*^reverse-score items.

**Anticipated obstacles academic aptitude struggles** I worry that	**Strongly disagree (%)**	**Disagree (%)**	**Neither agree/disagree (%)**	**Agree (%)**	**Strongly agree (%)**
^*^I will struggle with the workload	0	11.7	28.6	49.4	10.4
I struggle with the concept of academic teaching/learning	3.9	41.6	33.8	16.9	3.9
^*^the pace of teaching will be too fast	0	22.1	28.6	40.3	9.1
I lack the right study skills	7.9	28.6	36.4	22.1	5.2
I struggle with self-directed study	7.9	40.3	23.4	27.3	1.3
I will struggle with self-directed learning	10.4	37.7	20.8	28.6	2.6
I have chosen the wrong course	45.2	40.3	10.4	3.9	0
I may have made the wrong decision to go to university	46.8	35.1	15.6	2.6	0
**Other Struggles**					
I worry that					
^*^I will have financial difficulties	5.2	36.4	13	33.8	11.7
^*^I will suffer from examination anxiety	3.9	18.2	20.8	42.9	14.3
^*^there will be a lack of personal support from lecturers	5.2	40.3	23.4	29.9	1.3
I will be missing my family	27.3	16.9	11.7	39	5.2
I lack confidence	11.7	22.1	26	31.2	9.1
my family does not support me	66.2	29.9	2.6	1.3	0
I find it difficult to cope with being away from home	44.2	23.4	18.2	10.4	3.9

Less than 50% of students expected teaching to be different to what they experienced during A-levels, or at college, as seen in their responses to the statement on “lectures will be more informal than at school/college.” About 20% of students expected that lecturers would give extensive notes, however, between 75–90% of students expected having to be in charge of their own study habits (including note-taking, regular lecture attendance, group work, etc., see Table 4).

**Table 4:** Summary of endorsement of items presented for *Teaching Expectations* (in percentages); ^*^reverse-score items.

**Expectations of lecturers being facilitative** My expectations about attending university are that	**Strongly disagree (%)**	**Disagree (%)**	**Neither agree/disagree (%)**	**Agree (%)**	**Strongly agree (%)**
lectures will be more informal than at school/college	7.9	22.1	20.8	41.6	7.9
I will have to take care of my own notes	0	1.3	1.3	57.1	40.3
^*^I will not be required to attend classes	23.4	45.5	18.2	10.4	2.6
I will have to do a lot of independent learning	2.6	0	0	33.8	63.6
there will be a lot of group-work	1.3	3.9	27.3	63.6	3.9
I will be able to partake in research	1.3	1.3	5.2	70.1	22.1
**Expectations of lectures being dictative**					
My expectations about attending university are that					
lecturers give extensive written notes	9.1	37.7	31.2	18.2	3.9
lecturers will dictate their notes	5.2	23.4	26	44.2	1.3
^*^I will have to attend all classes	0	7.9	16.9	48.1	27.3
there will be too many assessments	1.3	15.6	44.2	35.1	3.9
it will be difficult to balance study and work commitments	0	20.8	35.1	35.1	9.1
**Expectations of lectures being easy; university not being different from high-school**					
My expectations about attending university are that					
I will do fine if I just pay attention in class	7.9	40.3	23.4	23.4	5.2
^*^I will do fine even if I do not go to class	48.1	39	10.4	1.3	1.3
I will do fine as long as I do all required reading	2.6	16.9	20.8	48.1	11.7
there will not be many assessments	9.1	53.3	33.8	3.9	0

### Student expectations questionnaire—cluster analysis

The numbers of clusters were predetermined to be 3—this was based on an initial Agglomerative Clustering method (squared Euclidean Distance). Initial cluster centers were then evaluated based on this sampling. The minimum distance between an assigned case and a cluster was observed to be 0; the maximum distance was 10. Final cluster centers were then generated as the mean for each variable within each final cluster. Final cluster centers reflect the characteristics of the *typical* case for each cluster. When assessing the cluster membership for students it emerged that only one student was assigned to cluster 2, 36 students were assigned to cluster 1 and 37 students were assigned to cluster 3. Three students remained unassigned.

Re-calculating the cluster analysis, forcing a decision between two-cluster assignments, resulted in 30 students being assigned to cluster 1 and 44 students being assigned to cluster 2, leaving the same three students unassigned. Results of the forced cluster analysis (presented in Table 5) indicate that, overall, the clusters are not too different from each other: Students in Cluster 1 endorsed *Academic Struggles, Expect Dictative (Information-Transmission) Teaching*, and *Other Struggles* less than students in Cluster 2, but students in Cluster 1 endorse *Lack Of Other Opportunities* more than students in Cluster 2.

**Table 5:** Results from “forced” Cluster Analysis.

	**Cluster**	
	**1**	**2**
**FINAL CLUSTER CENTERS**		
Academic ambition	17.17	16.11
Lack of other opportunities	15.60	13.84
Social factors	6.67	7.50
Perceived status and expectations	11.90	11.11
Academic struggles	16.80	21.66
Other struggles	16.77	18.93
Expect facilitative	24.40	23.07
Expect dictative	12.83	15.45
Expect easy	12.77	12.93

*Subscales and their themes are divided by shading of white to gray. Final cluster centers are computed as the mean for each variable within each final cluster*.

The findings from the ANOVA (here used in terms of a dispersion analysis of clustering results) allow assessment of the differences between F-ratios and, therefore, the role of different mean variables in the forming of the clusters. Findings from the ANOVA illustrate that *Academic Struggles, Expect Dictative (Information-Transmission) Teaching*, i.e., lecturer-focused teaching, *Lack of Other Opportunities*, and *Other Struggles* exerted the greatest influence in forming the clusters. Medium influence was exerted by *Expect Facilitative (Concept-Changing) Teaching*, i.e., student-focused teaching, *Perceived Status and Social/Parental Expectations* and other *Social Factors*. The least influence was exerted by *Academic Ambition* and *Expect Similarity to College/High-School Teaching*. The order of influences, and the associated *F*-values and significances, are summarized in Table 6.

**Table 6:** Summary of results from the dispersion analysis.

	***F***	***p*-value**
Academic struggles	82.3	0.001
Expect dictative (information-transmission) teaching	25.7	0.001
Lack of other opportunities	22.2	0.001
Other struggles	14.3	0.001
Expect facilitative (concept-changing) teaching	5.3	0.02
Perceived status and social/parental expectations	4.9	0.03
Social factors	4.8	0.03
Academic ambition	4.1	0.05
Expect similarity to college/high-school teaching	0.1	0.7

*Subscales and their themes are divided by shading of white to grey. Large F-values indicate greatest separation between clusters*.

### Student expectations questionnaire—exploratory analysis of age differences

To assess if age of student would have an effect on the scoring of the questionnaire we compared scores of those students who were under the age of 20 (*n* = 69) and those who were aged 20 and above (*n* = 8), using a Mann–Whitney *U*-test. This revealed a significant difference between groups, only with regards to the expectation of dictative, i.e., information transmission/teacher-focused teaching, *Z* = −1.9, *p* = 0.05. Here, those aged 18–19 years scored higher, meaning they “agreed” or “strongly agreed” to items like “*lecturers give extensive written notes”* and/or “*lecturers will dictate their notes”* than students who are aged 20 years or older (Table 7).

**Table 7:** Summary of results for Group differences when comparing students aged 18–19 vs. 20 years and over.

	**18–19 years old (*n* = 69)**		**20 years and over (*n* = 8)**	
	**Mean**	***SD***	**Mean**	***SD***
Academic ambition	16.8	1.9	15.1	3.8
Lack of other opportunities	14.7	1.8	13.8	1.8
Social factors	7.1	1.6	7.8	1.8
Perceived status and social/parental expectations	11.4	1.6	10.9	2.0
Academic struggles	19.8	3.2	18.6	3.7
Other struggles	18.0	2.7	16.9	3.0
Expect facilitative (concept-changing) teaching	23.5	2.5	24.5	2.3
Expect dictative (information-transmission) teaching^*^	14.6	2.4	12.6	2.8
Expect similarity to college/high-school teaching	12.7	2.0	13.8	1.8

* *Difference is significant, p = 0.05; n = number of participants; SD = Standard Deviation*.

### Approaches to teaching inventory (lecturers)

Overall, lecturers scored significantly higher on the CCSF scale (mean = 29.0; *SD* = 6.0; Range = 22; Min:Max = 15:37) than the ITTF of the ATI, (mean = 23.2; *SD* = 6.7; Range = 26; Min:Max = 10:36) scale, *t*_(19)_ = 2.4, *p* = 0.03. This means they adopt a concept-changing, student-focused approach over an information-transmitting, teacher-focused approach. Follow-up analysis on the intention subscales and strategy subscales of the CCSF and the ITTF scales supported the overall findings: significantly higher scores were revealed for the CCSF-strategy subscale (mean = 13.8, *SD* = 3.9; Range = 14; Min:Max = 6:20) relative to the ITTF-strategy subscale (mean = 10.8, *SD* = 3.5; Range = 12; Min:Max = 5:17), *t*_(19)_ = 2.3, *p* = 0.03; and trend-significant differences were shown for the intention subscales, *t*_(19)_ = 1.9, *p* = 0.07, with higher scores being reported for the CCSF-intention subscale (mean = 15.2, *SD* = 3.4; Range = 12; Min:Max = 7:19) relative to the ITTF-intention subscale (mean = 12.5, *SD* = 4.0; Range = 15; Min:Max = 5:20; Figure 1).

**Figure 1:**
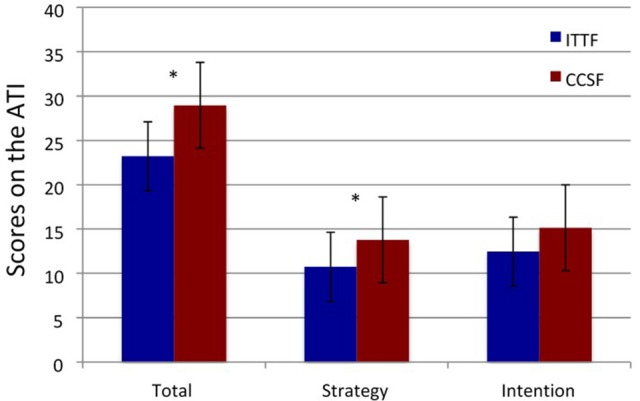
Score on ATI, showing the difference between ITTF (in blue) and CCSF (in red) total scores and on the Intention and Strategy subscales. ^*^Indicates significant differences between the scores.

### Approaches to teaching inventory—correlations with teaching experience

**Figure 2:**
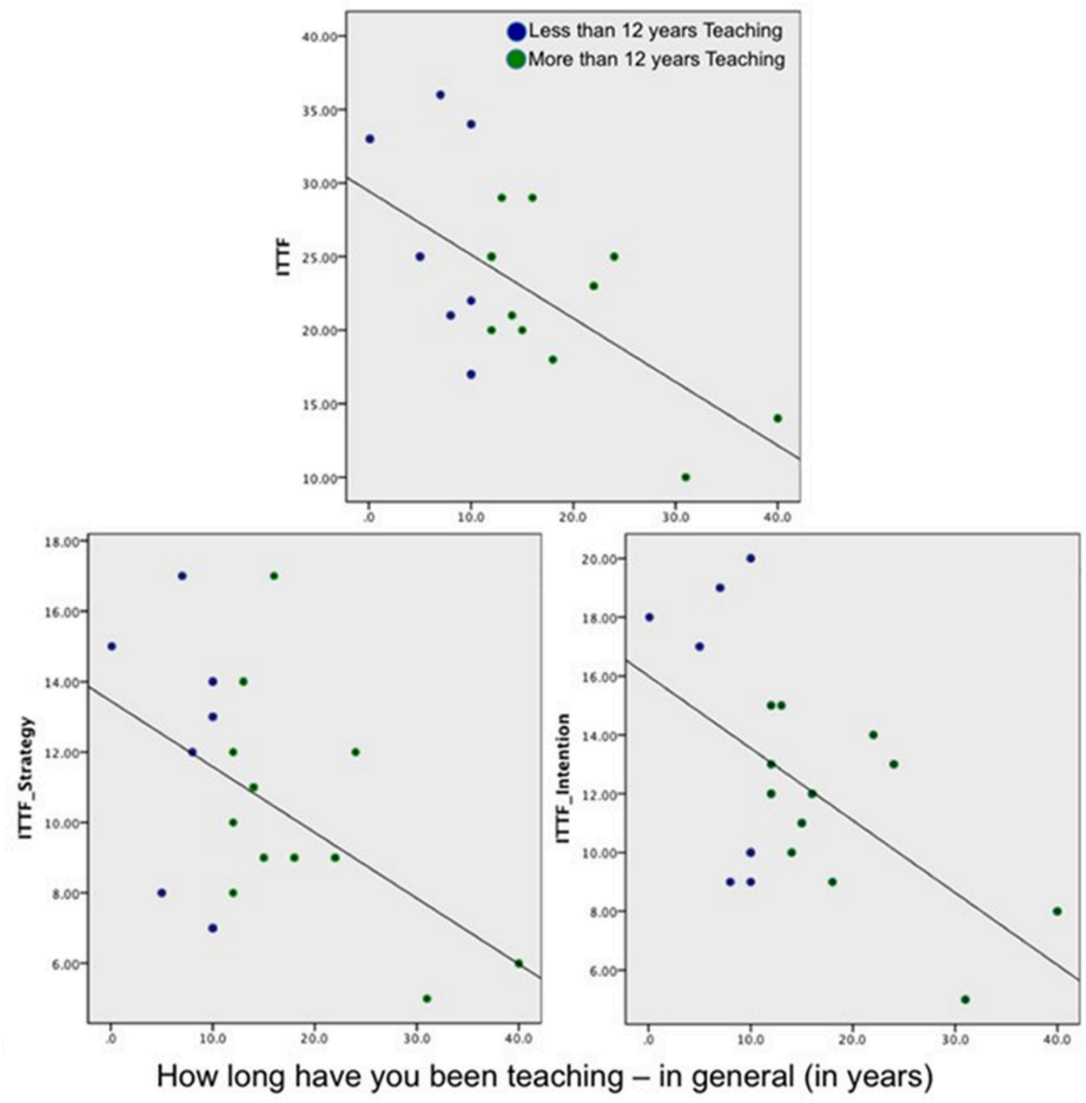
Correlations between years of teaching experience and scores on the ITTF scales.

When assessing the association between ATI scales and teaching experiences, a significant negative correlation was revealed between ITTF and years of teaching in general (see Figure 2), *r* = −0.6; *p* = 0.006, indicating that those who have been teaching fewer years endorsed approaches that are more teacher-focused and information-transmitting than their colleagues who have been teaching longer. This was further supported by significant and near-significant negative correlations between the ITTF subscales: ITTF-intentions subscale: *r* = −0.6, *p* = 0.009, and ITTF-strategy subscale: *r* = −0.5, *r* = 0.03 (see Figure 2).

Furthermore, assessing the associations between the lecturers' scores on the ATI measures and which year students are taught in (first year, second year, final year) revealed several significant relationships (see Figure 2): Significant and near-significant negative correlations were observed between year in which students are taught and lecturers scores on the ITTF (*r* = −0.6, *p* = 0.01) and scores on the ITTF-intention and ITTF-strategy subscales (*r* = −0.45 *p* = 0.05; *r* = −0.53, *p* = 0.02). Near-significant positive correlations were observed between year in which students are taught and lecturers' scores on the CCSF (*r* = +0.5, *p* = 0.04) and scores on the CCSF-Strategy subscale (*r* = +0.5, *r* = 0.02).

### Expectation of student engagement—group differences

Assessing differences in endorsements of positive and/or negative expectations of student engagement when comparing responses of lecturers who have been teaching longer (i.e., 12 years or more) and those who have been teaching 12 years or less, revealed a significant difference with regards to endorsement of negative expectations of student engagement: Lecturers with less teaching experience selected significantly more items (mean = 2.1; *SD* = 2.0) than lecturers with more teaching experience (mean = 0.5; *SD* = 0.7); *F*_(1, 18)_ = 7.2, *p* = 0.02. No significant differences were reported for endorsing positive expectations of student engagement.

Assessment of differences in endorsements of positive and/or negative expectations of student engagement when comparing responses of lecturers who teach first year vs. those who teach second year and above, yielded no significant group differences.

## Discussion

This study aimed to assess what incoming students and lecturers expect of learning and teaching at university. It was observed that, overall students, had relatively realistic expectations of university. For example, they viewed enrolling at university as helpful for making future career decisions, and the majority of students (over 75%) expected to be in charge of their own study habits. Less than 50% of students expected that teaching would be different at university than at secondary school—a finding in line with previous research (e.g., Cook and Leckey, 1999; Lowe and Cook, 2003). Approximately 60% of students expected to be struggling with the anticipated workload and nearly 50% of students anticipated that the pace at which teaching and learning takes place would be too fast. Emotional and financial struggles were anticipated by over 40% of students. This study shows consistency with previous findings such as those by Cook and Leckey (1999) and Lowe and Cook (2003).

### Student expectations

Cluster analyses, following the initial identification of students' endorsements of expectations (see Tables 2–4) revealed two independent clusters of students, showing that those who formed Cluster 2 were less assured of their own, independent learning. These students endorsed *Expect Dictative (Information-Transmission) Teaching* (i.e., information-transmitting, teacher focused approach to learning and teaching) as well as *Other Struggles* more often than the students who formed Cluster 1. Cluster 2 may have been comprised of students who enroll into university straight out of secondary school, anticipating little difference to the style of teaching they had encountered before (Lowe and Cook, 2003). These students would also expect to struggle more with the workload, the teaching pace and with studying more independently. Students in Cluster 2 also anticipated more struggles, both *Academic* and *Other*, such as emotional problems or financial hardship. Students forming Cluster 1, on the other hand, endorsed *Lack of Other Possibilities* as a reason for attending university more than the students forming Cluster 2. These students may be the ones who opted for a university education due to the fact that alternatives, such as going into vocational training or decent-paying jobs, are more constrained nowadays; even entry-level jobs often requiring at least a baccalaureate education (Wells et al., 2013).

Exploratory analyses assessed if age impacted the scoring of the questionnaire. It revealed a difference between those who are 18 and 19 years and those who are 20 years and older. Younger students expected more *information-transmitting teacher-focused approaches* than did older students. For example younger students more likely to expect that lecturers would give extensive written notes. Similar observations were reported by Lowe and Cook (2003); although their student sample was divided, with one group of students expecting much more detailed notes than they received, but the other group reporting they were, in fact, receiving more detailed lecture notes than they had anticipated. However, as Lowe and Cook (2003) studied students enrolled across different university courses the observed differences may have been related to the specific subject area those students were studying. The greater tendency of the younger students to expect this teaching approach may be because they have just left secondary school, whereas older students may have taken a Gap-Year, attended preparation courses for university, or joined university from the workforce. These experiences may have altered their expectations of what type of teaching to expect, or, more importantly, of their own abilities to study, learn and problem-solve independently.

The identified clusters, were also influenced by students' expectations of *Perceived Status* and *Parental Expectation*s, but to a lesser extent. This might relate to the perceived impact of parents' own educational attainments on students' academic expectations. For example, Cohen (1987) showed that parental influences had an impact on educational aspirations, as well as educational attainments. It has been argued that parental aspirations and expectations might possibly exert even more of an influence than status attainment or peer pressure (Kandel, 1978).

### Staff expectations

#### Approaches to teaching inventory

Overall, lecturers scored significantly higher on the concept-changing student-focused (CCSF) scale than the information-transmitting teacher-focused (ITTF) scale of the ATI (Trigwell and Prosser, 2004), indicating that lecturers more often adopt a student-focused approach in order to facilitate conceptual change in students with regard to the module they teach, rather than engaging in a more shallow, information-transmitting approach. The significant negative correlation observed between ITTF and its subscales and years of teaching showed that those with fewer years of teaching endorsed approaches that are more teacher-focused and information-transmitting. These findings also indicate that teachers tend to evaluate their teaching expectations in the context of their teaching experiences, as those with more teaching experiences endorsed such approaches less.

Significant and trend-significant negative correlations were observed between year in which students are taught and lecturers' scores on the ITTF and scores on the ITTF-intention and ITTF-strategy subscales. Trend-significant positive correlations were observed between year in which students are taught and lecturers' scores on the CCSF and scores on the CCSF-Strategy subscale. These findings indicate that there are associations between the approaches lecturers take (i.e., concept changing/student-focused vs. information-transmitting/teacher focused) and which year students are studying in. The nature of these associations (negative/positive) indicates that, for students in the earlier years of study, lecturers tend to endorse more information-transmission (teacher-focused) approaches. On the other hand, increasing years at university and cumulative learning experiences, the scores on the ITTF and its subscales decrease, meaning that lecturers endorse these teaching approaches less often. The positive relationships between the CCSF and the years in which students are studying supports these findings, as these associations show that lecturers tend to increase the student-focused, concept changing approaches in later years of study. This is in line with literature showing that lecturers adapt their approaches to teaching in responses to students' requests but also in response to students' learning and achievements (Trigwell and Prosser, 1993, 2004; Prosser and Trigwell, 1999). Such development is important to prepare students for post-graduate studies or for employment. It also shows that such development takes into account that students who come to university straight from A-levels, or college and who, as shown here, expect a teaching style more reflective of one they are used to, have an opportunity to gradually develop a more independent learning style.

### Expectation of student engagement

We observed that lecturers who teach students in second-year and/or above would show a larger number of positive learning endorsements relative to lecturers who taught first year students. However, Fraser and Killen (2003) showed that lecturers actually expected students to be independent, self-motivated and self-efficient right from the beginning of their university degree, a finding which is in-part supported by our current observations. Lecturers endorsed positive student engagement related to lecture attendance and participation in lectures far more than negative engagements (e.g., disruptive behavior, leaving early). Positive engagement with the university culture and a lecture, rather than a classroom, environment was endorsed by students, who also recognized regular attendance at lectures would be expected of them when at university. This seems to contradict findings by Fraser and Killen (2003), who reported that students undervalued the importance of regular lecture attendance.

### Application to students' university experience

A mis-match between students' and lecturers' academic expectations may result in communication break-down or to uncertainties about their respective roles. For example, students may feel that there is little that they can do to succeed and lecturers may not be aware of how they can improve the situation. In the long-term this could impair effective teaching and pedagogy and might lead to decreased student satisfaction, poor academic performance, and increased dropout rate (Fraser and Killen, 2003).

Current findings suggest a potential for common understanding, e.g., both students and lecturers endorsed regular lecture attendance and positive engagement during lectures as being expected when studying at university. This is in line with previous research (e.g., Crisp et al., 2009), but also contradictory to observed trends at university which have seen increasing rates of non-attendance at lectures (Cleary-Holdforth, 2007; Field, 2012) and a need for provisions such as online lecture repositories and increasing e-resources being requested by students. Yet, there are also quite significant differences, suggesting disparate views of what a successful academic career, or successful academic progression, means. Talbot (1990) reported that the most influential personality traits (in relation to academic persistence and achievement) appeared to be intrinsic motivation and students' level of cognitive categorization. The importance of understanding whether or not there is a mis-match between the expectations that students hold of university teaching and learning, and the expectations that staff have of students is related to the fact that the majority of students who end up dropping out of university do so in year 1, and most likely at the end of term 1, or the beginning of term 2 (Ozga and Sukhnandal, 1998).

A HEFCE report (HEFCE, 2017) shows that retention rates in 2011–2012 were about 6.6%; higher drop-out rates (non-continuation rates) were observed for mature students (and those in age-brackets of 21–24 and 25 and over). It appears as if males are more likely to drop-out than females, hence it may be important to look at gender differences with regards to expectations. The low number of males recruited in this study does not, however, allow for a rigorous assessment of gender differences. There is a documented “gender gap” in attending university, in fact, data from acceptance and enrollment rates in 2015 showed that the entry rate for female students aged 18 grew twice as fast as that for males, meaning that females are 35% more likely to enter university than males (UCAS, 2016). Previously, different academic expectations between males and females have also been reported (Wells et al., 2013); this aspect should be further addressed in future.

Students are particularly vulnerable at the beginning of the course; hence they may require more support. Research has shown that the introduction of orientation courses has resulted in higher academic achievement and lower drop-out rates (Wilke and Kuckuck, 1989). The identification of students at-risk of failure, but also assessments of students' expectations and their satisfaction as well as offering tutoring services and study skills development programs have proven to be successful in maintaining, if not improving, retention rates (Cook and Leckey, 1999).

Therefore, considering the different perspectives would help in attempting to narrow the gap between discrepant expectations. Helping students understand the apparent changes between studying at secondary school and studying at university would allow for more realistic expectations from the beginning, including a reduction in anxiety and a potential for better academic success. From a lecturers' perspective, helping students to become more aware of, and to understand, effective, and progressive learning habits and learning environments (Fraser and Killen, 2003) would increase their academic potential and ensure more successful degree completions.

Specifically, younger students which in this study made up the majority of the sample, expected teaching to be much more information-transmitting, facilitating the more shallow learning approaches they are familiar with, or successfully applied, at college. Recent recognition of “*Life-long Learning”* aims to increases the number of mature students into higher education; however, differences with regards to student characteristics, e.g., students' prior experiences and circumstances, would need to be considered more closely. Nonetheless, the number of new undergraduates in the UK reached record levels in 2015, with UCAS reports revealing increasing number of students from disadvantaged backgrounds, mature students as well as students from ethnic minorities and those who are first-generation of attending university entering higher education. To ensure their retention, progress and ultimately success is reliant upon closing the gap between the differing expectations hold amongst students and lecturers.

### Limitations

Students voluntarily filled out the questionnaires, rather than it being a compulsory requirement for a course, for example. It has thus to be considered that the sample is likely biased toward more engaged and proactive students in the first place. No record of whether students would be considered to be of a traditional, compared to non-traditional, background with regards to university education was obtained, a fact that likely could have impacted results. Although we recorded if students were entering their first-ever degree course, or if they had previously entered a course, the numbers were too discrepant in order to compare them in any meaningful way. In future, university education background, i.e., traditional vs. non-traditional, should be recorded as there may be differences in expectations between these groups of students. It might be useful to more actively recruit those who had previously entered a degree course, and to compare their expectations of university teaching and learning against those who had never entered a degree programme before.

Overall, the sample size is modest, and given that the sample was obtained primarily from only one programme (BSc Psychology)—which traditionally has a very imbalanced male:female ratio—in future, studies should recruit across different university programmes to balance the number of male and female students who are being asked about their expectations of university. The imbalance in male:female ratio could confound findings, given the previously discussed gender differences with regards to academic expectations (Seifert et al., 2010; Wells et al., 2013). Recruitment of a more evenly balanced sample of male and females could be arranged by assessing degree courses that may be unevenly represented across genders (e.g., comparing Psychology and Engineering).

In this sample, the number of students who were aged 20 and above, and are thus regarded as mature students, was rather small (*n* = 8). Differences between this cohort and the younger student cohort should be viewed with caution. Future research, however, should attempt to increase the number of mature students in order to assess such differences in detail.

## Conclusion

Higher education is an extremely important and life-changing time for most students; students invest not only financially, but also emotionally as well as time and effort. Therefore, ensuring that students make the most of their university experience, and leave university with the best degree possible requires clear communication of the expectations that both parties, students and lecturers, have of each other. What can be drawn from this study is that there *remains a need* to more clearly communicate these mutual expectations. From a lecturer's perspective, reiterating the *active* and *self-governing* role that students need to play in their university education might resolve in students being more aware of the fact that they would need to accept full responsibility for their own academic success and acknowledge that their lecturers are only one of many resources for achieving success. Students need to be made aware of the fact that they need to monitor their own progress toward completing their degree (Tinto, 1995). Furthermore, it needs to be acknowledged that students and lecturers have *joint responsibility* for student success: a first stage in accepting such responsibility would be to gain a better understanding of the complex processes that seem to influence students' academic success. Differences in student and lecturer perception and expectation make it difficult to appropriately assess learning and teaching. Future research should therefore attempt to further integrate students' expectations about the factors that may influence their success with their actual performance (Fraser and Killen, 2003).

## Author contributions

SH: designed and conducted the study (data collection and analysis) and wrote the initial draft of the manuscript; NR: conducted a literature search and wrote the second draft of the manuscript. Both authors contributed to the final draft.

### Conflict of interest statement

The authors declare that the research was conducted in the absence of any commercial or financial relationships that could be construed as a potential conflict of interest.
